# Nontraumatic Epithelial Ingrowth 15 Years Post Laser In Situ Keratomileusis

**DOI:** 10.1155/2019/5270636

**Published:** 2019-10-07

**Authors:** Joanna S. Saade, Baha' Noureddin, Shady T. Awwad

**Affiliations:** The American University of Beirut Medical Center, Cairo Street, Beirut, Lebanon

## Abstract

*Purpose. *Epithelial ingrowth occurring many years after primary Laser in Situ Keratomileusis (LASIK) without a preceding traumatic event is very rare. *Case Report.* We describe the case of a 61-year-old woman with epithelial ingrowth in her right eye 15 years after primary LASIK. She presented with right eye redness, pain, and decreased vision and denied any preceding trauma. An epithelial cells' tract was visible on Optical Coherence Tomography. Conservative treatment lead to the stabilization of the epithelial nests. *Discussion.* Epithelial ingrowth can occur many years after LASIK and may be due to a microtrauma to the edge of the flap.

## 1. Introduction

Epithelial ingrowth is a rare complication of Laser in Situ Keratomileusis (LASIK) refractive surgery. It is defined as the migration of surface epithelial cells under the surgical flap [1]. We report the case of a 61-year-old woman presenting with nontraumatic epithelial ingrowth occurring 15 years post-LASIK surgery.

## 2. Case Report

A 61-year-old woman presented to the eye clinic at the American University of Beirut Medical Center (AUBMC) complaining of redness, decrease in vision and pain in her right eye. It had started 5 days ago following a soft contact lens (CL) application for a few hours. She had never worn a contact lens before. She self-medicated with an eye drop containing tobramycin and dexamethasone, 1 drop four times daily, and reported slight improvement. Past medical history review was significant for a LASIK procedure to correct myopia 15 years ago not necessitating retreatment. On her last follow-up exam 3 months prior to presentation, her corrected distance visual acuity (CDVA) was 20/20 in her right eye, wearing +2.25 −1.00 × 120. At the time, the corneal exam featured a clear, well placed flap in both eyes without the presence of any epithelial ingrowth.

**Figure 1:**
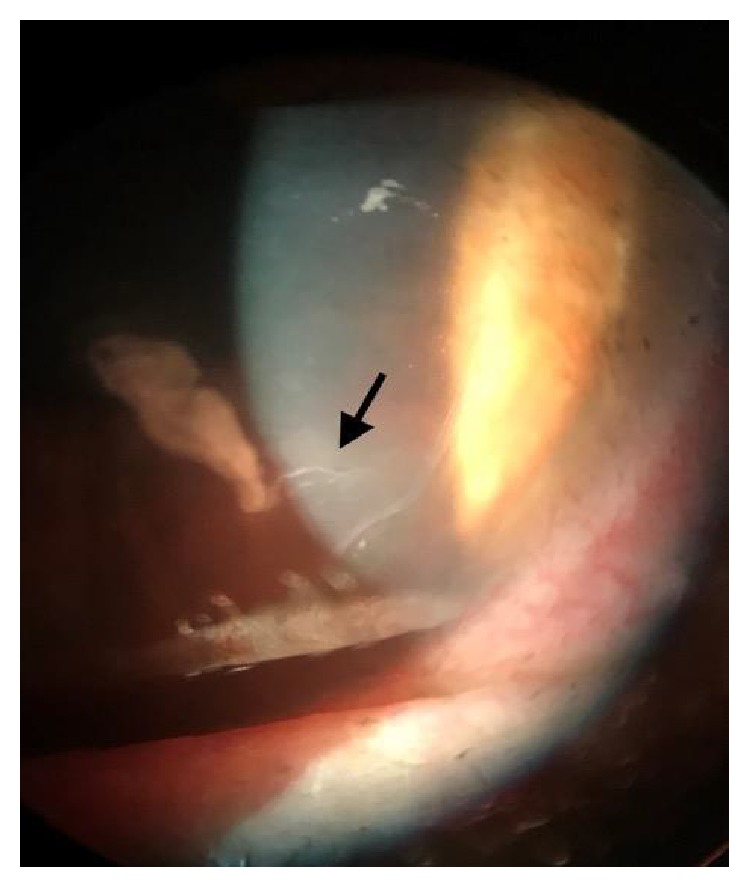
Anterior segment photo of the epithelial ingrowth tract (arrow) at the time of presentation.

**Figure 2:**
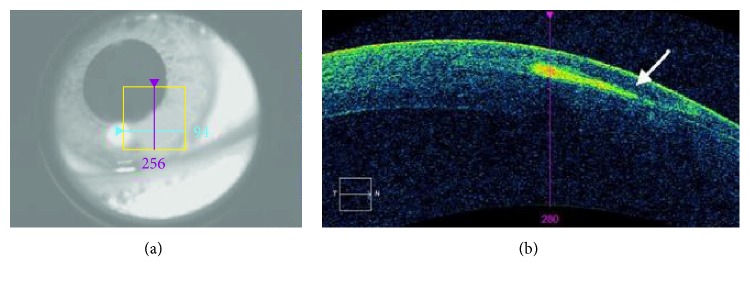
(a) Corneal area imaged by the Optical Coherence Tomography (OCT). (b) The OCT showing the epithelial tract under the flap at 5 o'clock (arrow).

On presentation, examination of the right eye revealed a CDVA of 20/20-1. The flap configuration indicated that a microkeratome was used at the time of her surgery 15 years ago. Evidence of a new apparent large sheet of confluent opacity measuring 3 mm, surrounded by haze and stromal edema was noted 2 mm from the edge of the flap at 5 o'clock. There was no epithelial defect, however, the edge of the flap was slightly retracted at 5 o'clock with mild fluorescein pooling (Figure 1). The left eye exam was unremarkable with a CDVA of 20/20 and a visible superiorly hinged flap. No epithelial basement dystrophy was seen in both eyes. Optical Coherence Tomography of the anterior segment using the Cirrhus OCT (Carl Zeiss Meditec, Dublin, California, USA) showed an epithelial tract beneath the flap in her right eye (Figure 2).

The diagnosis of epithelial ingrowth was discussed with the patient and flap lifting for debridement was advised but the patient preferred waiting. Her drops were discontinued and Moxifloxacin was initiated, one drop four times daily to cover broadly possible bacterial super-infection.

Two weeks later, the stromal edema resolved while the white epithelial nests persisted, and no overlying melting was seen.

## 3. Discussion

Epithelial ingrowth following primary LASIK is a reported complication in 1−3.9% of cases when using a microkeratome [2]. Ninety percent of those cases are detected within 2 months following the procedure [1]. It usually occurs when the flap edge, integral for wound healing, is disrupted. Studies conducted on rabbit corneas showed that following a microkeratome injury, healing occurs at the periphery of the wound sparing the central optical zone [3].

LASIK retreatment, diabetes mellitus as well as epithelial injury have been shown to increase the risk of epithelial ingrowth [4].

It is possible that CL wear lead to the epithelial ingrowth. However, the more likely scenario is that a slow subtle epithelial ingrowth has been growing progressively at some point post the LASIK surgery and was missed on previous exams. A micro-trauma caused by the use of CL most likely precipitated an infection/inflammation that made the condition clinically significant leading the patient to seek medical attention. This is supported by the quick response to the steroid and antibiotic drops while the epithelial nests persisted on future follow ups.

To the best of our knowledge, this report is amongst the first to describe a nontraumatic clinically significant epithelial ingrowth occurring 15 years post-LASIK. Only one report describes epithelial ingrowth without flap dislocation years following LASIK, but it was secondary to trauma with a twig [5].

## 4. Conclusion

Epithelial ingrowth can occur several years after LASIK without a preceding traumatic event. Microtrauma to the flap edge might be all what it takes to uncover or possibly precipitate this often-challenging condition.

## Additional Points

*Authorship*. All authors attest that they meet the current ICMJE criteria for Authorship.

## References

[1] Wang M. Y., Maloney R. K. (2000). Epithelial ingrowth after laser in situ keratomileusis. *American Journal of Ophthalmology*.

[2] Ting D. S. J., Srinivasan S., Danjoux J.-P. (2018). Epithelial ingrowth following laser in situ keratomileusis (LASIK): prevalence, risk factors, management and visual outcomes. *BMJ Open Ophthalmology*.

[3] Pérez-Santonja J. J., Linna T. U., Tervo K. M., Sakla H. F., Alió y Sanz J. L., Tervo T. M. (1998). Corneal wound healing after laser in situ keratomileusis in rabbits. *Journal of Refractive Surgery*.

[4] Jabbur N. S., Chicani C. F., Kuo I. C., O’Brien T. P. (2004). Risk factors in interface epithelialization after laser in situ keratomileusis. *Journal of Refractive Surgery*.

[5] Aboumerhi H., Shultz C., Erzurum S. A. (2015). Traumatic epithelial ingrowth despite nondisplaced lasik flap. *MOJ Clinical & Medical Case Reports*.

